# A primary human macrophage-enteroid co-culture model to investigate mucosal gut physiology and host-pathogen interactions

**DOI:** 10.1038/srep45270

**Published:** 2017-03-27

**Authors:** Gaelle Noel, Nicholas W. Baetz, Janet F. Staab, Mark Donowitz, Olga Kovbasnjuk, Marcela F. Pasetti, Nicholas C. Zachos

**Affiliations:** 1 Center for Vaccine Development, University of Maryland School of Medicine, Baltimore, MD, USA.; 2 Department of Medicine, Division of Gastroenterology and Hepatology, Johns Hopkins University School of Medicine, Baltimore, MD, USA.

## Abstract

Integration of the intestinal epithelium and the mucosal immune system is critical for gut homeostasis. The intestinal epithelium is a functional barrier that secludes luminal content, senses changes in the gut microenvironment, and releases immune regulators that signal underlying immune cells. However, interactions between epithelial and innate immune cells to maintain barrier integrity and prevent infection are complex and poorly understood. We developed and characterized a primary human macrophage-enteroid co-culture model for in-depth studies of epithelial and macrophage interactions. Human intestinal stem cell-derived enteroid monolayers co-cultured with human monocyte-derived macrophages were used to evaluate barrier function, cytokine secretion, and protein expression under basal conditions and following bacterial infection. Macrophages enhanced barrier function and maturity of enteroid monolayers as indicated by increased transepithelial electrical resistance and cell height. Communication between the epithelium and macrophages was demonstrated through morphological changes and cytokine production. Intraepithelial macrophage projections, efficient phagocytosis, and stabilized enteroid barrier function revealed a coordinated response to enterotoxigenic and enteropathogenic *E. coli* infections. In summary, we have established the first primary human macrophage-enteroid co-culture system, defined conditions that allow for a practical and reproducible culture model, and demonstrated its suitability to study gut physiology and host responses to enteric pathogens.

Coordinated interaction between the intestinal epithelium and immune cells is required to maintain proper barrier function and mucosal immunity that can prevent infection in the human gut^1^. Despite the development of several animal and human models proposed to study the cellular and molecular events occurring at the intestinal interface, there remains a need for a practical and reproducible *in vitro* model that employs human primary tissue to confirm and advance our current understanding of gut physiology and mucosal immunology^2^. Indeed, while animal models have contributed to our understanding of gut immunology, the biological differences between human and other mammalian species limit the relevance of the data generated through these systems^3^. *Ex vivo* experimentation using human intestinal mucosa is a viable approach but limited by tissue complexity (the system cannot be reduced to dissect interactions between discrete cell types), insufficient amount or quality of tissue available, and the need for repeated access to biopsy samples from human donors^4^. While models composed of human primary isolated intestinal and immune cell types have been used to interrogate interactions between specific cell types, the challenges regarding tissue availability (particularly from healthy individuals), poor tissue characterization, and short viability remain an obstacle that limits their use^5^,^6^. Immortalized stable human cell lines such as Caco-2, T-84, and HT-29 cells with one or more immune cell types in assorted co-culture formats^7^,^8^,^9^ have also been applied to address questions regarding epithelial-immune cell interactions and communication. The human neonatal small intestinal line H4 is an alternative non-transformed model of epithelial cells, although it is unclear whether these cells retain the characteristics of the intestinal tissue *in vivo*. It is also unlikely that one cell type would reflect its diverse composition and complexity^10^,^11^. Hence, the development of an epithelial-immune co-culture model derived entirely from primary human tissue that does not require fresh patient samples for each study and exhibits normal intestinal features is a necessary advancement for studying coordinated epithelial immune response under normal or pathogenic conditions^12^. Human enteroids provide an attractive framework for developing *in vitro* models of gut architecture and function using primary human tissue^13^,^14^,^15^,^16^. Enteroids and colonoids derived from LGR5^+^ stem cells or LGR5^+^-containing crypts from the small intestine and colon^17^, respectively, contain the four major human intestinal epithelial cell lineages with distinct roles in gut homeostasis and immune modulation (i.e. absorptive enterocytes, mucus-producing Goblet cells, hormone-producing enteroendocrine cells, and antimicrobial molecule-producing Paneth cells). Further, enteroids and colonoids recapitulate important aspects of human intestinal physiology^18^,^19^. Enteroid monolayers that allow experimental access to both apical and basolateral sides of the epithelial cells have been recently developed^20^,^21^. These monolayers provide a structure for basolateral addition of relevant cell types, such as immune cells, to interrogate specific physical interactions, paracrine communication, and molecular mechanisms underlying host responses to apical stimuli.

In this work, we describe the development and characterization of a macrophage-enteroid co-culture model consisting of human enteroid monolayers and human monocyte-derived macrophages (one the most prominent cell types participating in innate and adaptive host defenses) and its successful application to investigate intestinal epithelial and macrophage interactions, and their responses to enteric pathogens.

## Results

### Enteroid monolayers recapitulate features of human small intestinal physiology *ex vivo*

**Figure 1:**
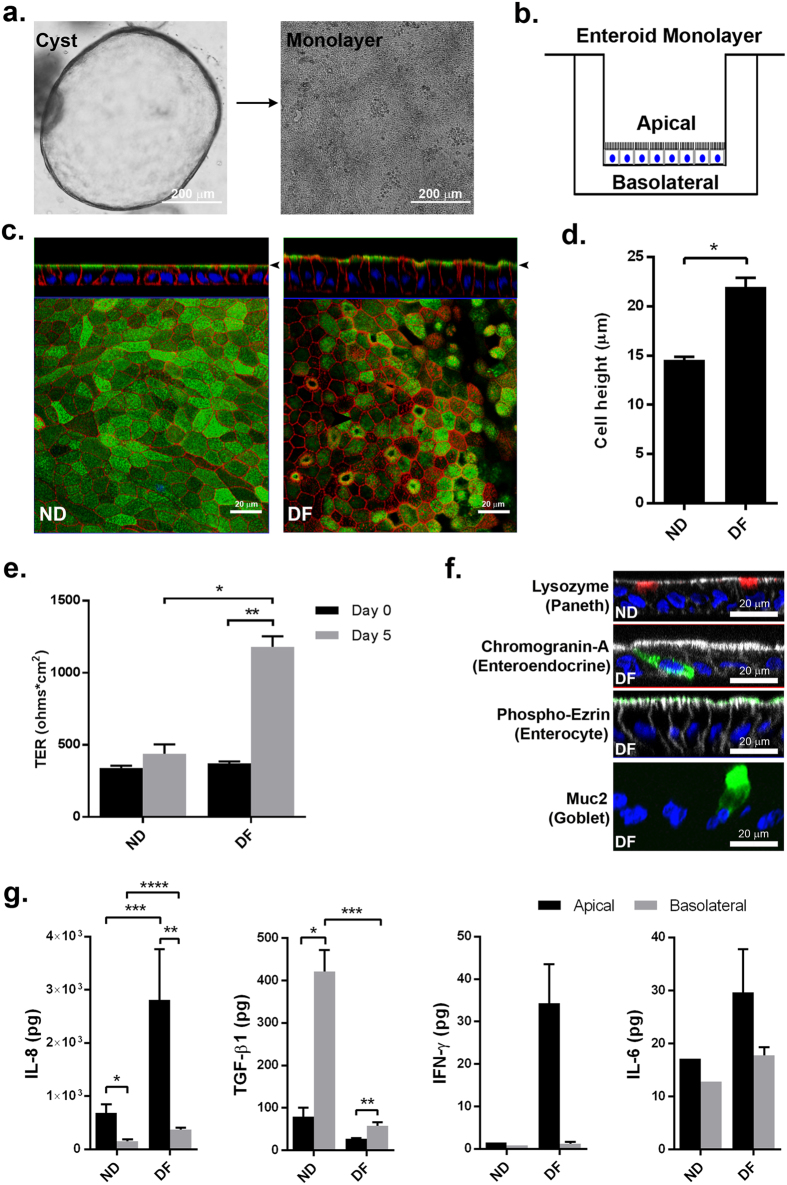
Human enteroid monolayers form mature intestinal epithelium *ex vivo.* (**a**) Human small intestinal enteroids cysts derived from biopsies (left panel) were grown as confluent epithelial monolayers (right panel) on permeable inserts. (**b**) Schematic representation of polarized enteroid monolayer established on permeable insert. (**c**) Non-differentiated (ND; left panel) and differentiated (DF; right panel) enteroid monolayers from small intestinal biopsies were visualized by confocal microscopy and showed polarized apical surfaces (wheat germ agglutinin [WGA]), green; indicated with black arrowheads in top panels). Mature microvilli were observed on the apical surface of DF enteroid monolayers (actin, red). XZ projection, top panels; XY projection, bottom panels; nuclei, blue. (**d**) Cell heights significantly increased in DF enteroid monolayers (*indicates *p* = 3.21 × 10^−21^). (**e**) TER increased upon 5 days of differentiation (*indicates *p* = 2.13 × 10^−6^; **indicates *p* = 4.96 × 10^−9^). (**f**) Paneth cells (lysozyme, red; top panel) were found in ND enteroid monolayers. Enteroendocrine cells (chromogranin-A, green), enterocytes (phospho-ezrin, green), and goblet cells (MUC2, green) were observed in DF enteroid monolayers. Nuclei, blue; actin, white. (**g**) Total amount of secreted cytokines released in the apical and basolateral media were quantified following 24 h of culture for IL-1β, IL-2, IL-4, IL-6, IL-8, IL-10, IL-12p70, IL-13 IFN-γ, and TNF-α, and 72 h of culture for TGF-β1, in ND and DF enteroid monolayer cultures. IL-8: *indicates *p* = 0.0132; **indicates *p* = 0.0010; ***indicates *p* = 0.0133; ****indicates *p* = 0.0009. TGF-β1: *indicates *p* = 0.0067; **indicates *p* = 0.0448; ***indicates *p* = 0.0018. Values are presented as Mean + SEM of at least three independent experiments involving enteroids generated from at least three individual donors with the exception of IFN-γ and IL-6 production in ND cultures that represent Mean +© SEM of two independent experiments with enteroids generated from two different donors (Fig. 1g).

In order to develop a primary human small intestinal model for the purpose of studying epithelial-macrophage interactions and signaling, we first established human enteroid monolayers according to previously described methods^21^ and examined their capacity to recreate a polarized epithelial barrier with the unique features of the normal human intestine. Enteroid cysts (three-dimensional [3D] structures) were seeded onto permeable membrane supports and grown to confluency (Fig. 1a,b) to generate monolayers (two-dimensional [2D] structures). The resulting monolayers allow for controllable access to both apical and basolateral surfaces of the intestinal epithelial cells (Fig. 1b). Previous studies established that the removal of Wnt3A from enteroid and colonoid cultures leads to the differentiation of LGR5^+^ stem cell-containing enteroid cultures (referred to as the non-differentiated [ND] state) into the distinct cell lineages present in the human intestinal epithelium generating the “villus-like” (referred to as differentiated [DF] state) epithelium^13^,^21^,^22^. In this study, enteroid monolayers, derived from duodenum and jejunum of multiple healthy donors, were maintained in either Wnt3A-containing non-differentiation media (NDM) or Wnt3A-free differentiation media (DFM)^23^ to examine the morphological and functional features of the ND and DF enteroid monolayers. Confocal microscopy of fluorescently labeled actin and wheat germ agglutinin (WGA; indicating glycocalyx-rich cell membrane) showed that both conditions resulted in an organized and polarized epithelium (Fig. 1c) consistent with previously published data^13^,^14^. Enteroid differentiation (five days in DFM) was associated with defined brush border formation demonstrated by apical actin labeling (Fig. 1c, right panel) and a significant increase in cell height when compared to the ND monolayers (Fig. 1d). DF, but not ND, monolayers also exhibited a significant increase in barrier function transepithelial electrical resistance (TER) (Fig. 1e). Immunofluorescent labeling of ND enteroid monolayers showed cellular characteristics of intestinal “crypts” evidenced by lysozyme-positive Paneth cells (Fig. 1f, upper panel), whereas the DF enteroid monolayers showed cellular characteristics of intestinal “villi” as evidenced by the presence of phospho-ezrin-positive enterocytes, chromogranin A-positive enteroendocrine cells, and Muc2-positive goblet cells (Fig. 1f). Paneth cells have a particularly long lifetime^24^ and were still present in DF enteroids, probably remnant from ND structures (data not shown).

To assess the ability of the enteroids to produce and secrete soluble immunological markers, we investigated the presence of 11 cytokines and chemokines in culture media exposed to the apical and basolateral compartments of enteroid monolayers. IL-1β, IL-2, IL-4, IL-10, IL-13, IL-12p70 and TNF-α were undetectable or present at very low levels (below 1 pg; see Supplementary Table S1). Interestingly, large amounts of TGF-β1 and IL-8 (Fig. 1g), two critical cytokines produced by human intestinal mucosa *in vivo*^25^, were produced by the small intestinal enteroid cultures. IFN-γ and IL-6 were produced at levels reaching 50 pg (Fig. 1g). All of them, with the exception of IL-6, were secreted in a polarized manner; higher levels of TGF-β1 were detected in the basolateral compartment, whereas IFN-γ and IL-8 were secreted mainly from the apical surface (Fig. 1g). In addition, while IL-6 did not seem to be influenced by differentiation state, IFN-γ and IL-8 were produced more abundantly by the DF “villus-like” enteroid as compared to the ND “crypt-like” counterpart, and the opposite was true for TGF-β1 production. Together, these results demonstrate that enteroid monolayers maintain appropriate barrier function and cell diversity in addition to secreting cytokines whose production can be spatially measured.

### Generation of mature human macrophages to establish a macrophage-enteroid co-culture system

**Figure 2:**
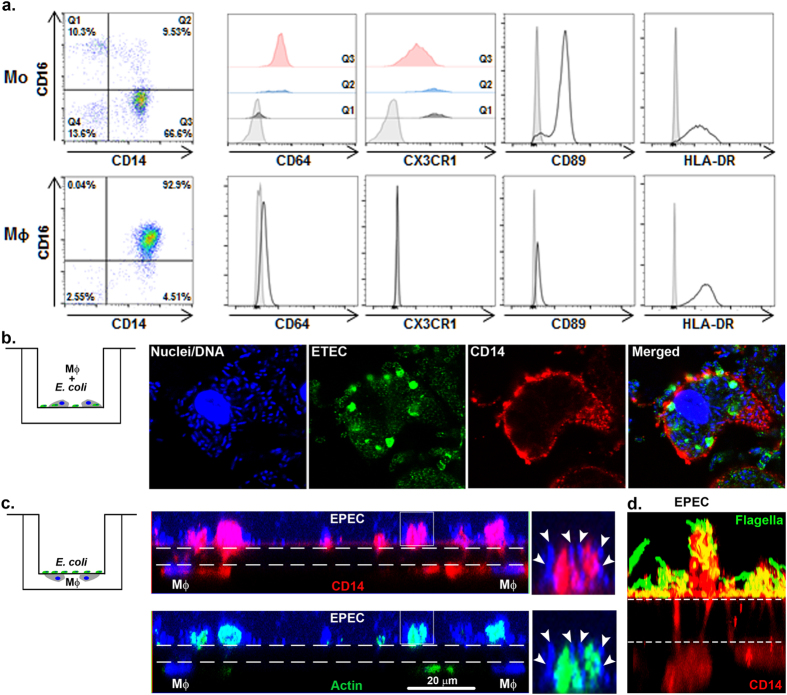
Human macrophages display immune phenotype and bacterial sensing. (**a**) Freshly isolated human monocytes (Mo; upper panels) and the derivative differentiated macrophages (MΦ; lower panels) were assessed by flow cytometry for surface expression of immune cell markers including CD14 and CD16 (shown as dot plots) and CD64, CX3CR1, CD89 and HLA-DR (shown as histograms). For Mo, the three main populations were reported as Q1, Q2 and Q3. (**b**) ETEC incubated with MΦ (schematic representation, left panel). Following overnight infection, bacteria were phagocytosed by MΦ. DNA, blue; ETEC, green; CD14 (MΦ), red. (**c**,**d**) MΦ were seeded underneath the filter with EPEC added onto the insert (schematic representation, left panel). MΦ extended projections across the 1.0 μm pore insert filter to reach EPEC, added on the opposite surface of the insert membrane. Dashed lines indicate the position of the 1.0 μm filter. (**c**) DNA, blue; actin, green; CD14, red; EPEC DNA, arrowheads. (**d**) EPEC flagella, green; CD14, red.

In order to investigate the interaction between intestinal epithelial and immune cells and their role in anti-microbial defenses, we developed, as an initial step, an enteroid monolayer culture system that included macrophages. Macrophages (derived from human peripheral blood monocytes) were selected as the first cell lineage to assemble with the intestinal epithelium because they provide a critical first line of innate host immune defense. To this end, monocytes (Mo) isolated from blood of three healthy donors were differentiated into primary mature but not activated macrophages. Three different monocyte subsets were identified by flow cytometry (i.e. Q1, Q2, and Q3; n = 8, including three different donors) on the basis of cell surface marker expression that included: a major CD14^+^ CD16^−^ CD64^+^ CX3CR1^low^ classical population (Q3; 76.3% ± 5.0), a CD14^−^ CD16^+^ CD64^−^ CX3CR1^high^ non-classical population (Q1; 9.0% ± 3.2), and a CD14 and CD16 double positive intermediate population (Q2; 9.1% ± 1.8) (Fig. 2a, upper panels). Following six days of differentiation in the presence of Macrophage Colony-Stimulating Factor (M-CSF), monocyte-derived macrophages (MΦ) were obtained that exhibited a homogeneous CD14^+^ CD16^low^ CD64^low^ CX3CR1^−^ phenotype (Fig. 2a, lower panels). Differentiated macrophages exhibited increased expression of antigen-presenting major histocompatibility complex (MHC) class II molecule, HLA-DR, while the monocyte surface marker, CD89, was downregulated as compared to freshly isolated monocytes. To confirm the functional capacity of these macrophages, they were exposed *in vitro* to two different strains of *Escherichia coli (E. coli*). Phagocytic activity of macrophages was demonstrated by internalization of enterotoxigenic *E. coli* (ETEC) incubated for 12 h with MΦ on the same side of the Transwell filter (Fig. 2b). Further evidence of phagocytic activity was provided by the internalization of enteropathogenic *E. coli* (EPEC) by MΦ seeded on the opposite side of the Transwell filter (Fig. 2c,d). This bacteria-MΦ compartmentalization was chosen to represent their location in the final macrophage-enteroid co-culture model. Macrophage phagocytic activity was associated with morphological changes such as their ability to generate projections that extended through 1.0 μm pore filters to physically interact with EPEC (Fig. 2c, middle and right panels) and its flagella (Fig. 2d). This interaction was facilitated via actin-dependent remodeling (Fig. 2c, lower panels). Together, these results demonstrated the successful generation of a homogeneous population of mature monocyte-derived macrophages that can be seeded underneath 1.0 μm pore filter inserts and respond to compartmentalized (top of the filter added) enteric bacterial pathogens.

### Establishment of macrophage-enteroid co-cultures and cross communication between macrophages and epithelial cells

**Figure 3:**
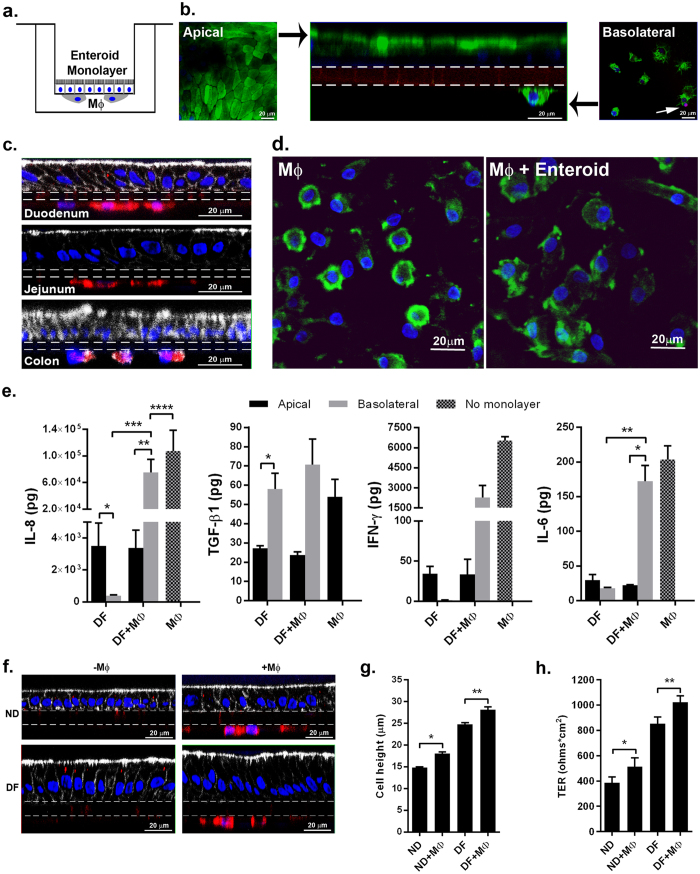
Human enteroid monolayers and monocyte-derived macrophages communicate in co-culture. (**a**) Schematic representation of human enteroid co-cultured with macrophages (MΦ). (**b**) Macrophage-enteroid co-culture showed polarized DF duodenal enteroid monolayer indicated by apical WGA labeling (green; left and middle panels) and MΦ facing the basolateral side of the epithelium (middle and right panels), both separated by a permeable membrane shown by dashed lines (middle panel). Propidium iodide (red, white arrow in right panel) did not label substantial number of cells. Nuclei, blue; XZ projection, middle panel; XY projection, left and right panels. (**c**) Macrophage-enteroid co-cultures were established with duodenal-, jejunal-, and colon-derived enteroid monolayers. Nuclei, blue; actin, white; CD14 (MΦ), red; filter, dashed lines. (**d**) MΦ cultured 24 h with DF enteroid monolayer (right panel) showed morphological changes when compared to MΦ cultured alone (left panel). Nuclei, blue; WGA, green. (**e**) IL-8, IFN-γ, and IL-6 secretion was increased in basolateral media of DF enteroid cultured 24 h with MΦ. TGF-β1 secreted in apical and basolateral media of 72 h macrophage-enteroid co-cultures was not significantly modulated by the addition of MΦ. IL-8: *indicates *p* = 0.0156; **indicates *p* = 0.0156; ***indicates *p* = 0.0156; ****indicates *p* = 0.0469. TGF-β1: *indicates *p* = 0.0448. IL-6: *indicates *p* = 0.0226; **indicates *p* = 0.0184. (**f**) Both ND and DF enteroid monolayers (left panels) could be cultured with MΦ (right panels). Filter, dashed lines; actin, white; nuclei, blue; CD14 (MΦ), red. (**g**) The presence of MΦ increased cell height in both ND and DF enteroid monolayers. *Indicates *p* = 1.06 × 10^−12^; **indicates *p* = 4.39 × 10^−5^. (**h**) Differentiation of the enteroid monolayers and the addition of MΦ increased TER. *Indicates *p* = 0.0391; **indicates *p* = 0.0105. Data are presented as Mean + SEM of at least three independent experiments involving enteroids generated from at least three individual donors.

A human macrophage-enteroid co-culture was established by culturing human enteroid monolayers and mature human macrophages, separated by a permeable membrane. Enteroids were seeded with the basolateral membrane facing the filter and accessible, through the filter pores, to macrophages seeded underneath the filter (Fig. 3a). Confocal imaging of the macrophage-enteroid co-culture showed fluorescently labeled WGA at the apical surface of DF enteroid monolayers derived from human duodenum and on adherent macrophages facing the basolateral side of the enteroid monolayer (Fig. 3b). The viability of these two cell types was confirmed by propidium iodide (PI) exclusion (Fig. 3b); less than 1% of the total filter-adhered macrophages were PI-positive after 24 h of co-culture (data not shown). The macrophage-enteroid model was successfully expanded to include DF enteroid monolayers developed from multiple sections of the human intestine (i.e. duodenum, jejunum and proximal colon) for the purpose of interrogating tissue specific questions (Fig. 3c). Macrophage-enteroid communication was examined using small intestinal DF enteroid monolayers (developed from duodenum and jejunum). Macrophages co-cultured with DF enteroid monolayers exhibited a distinct morphology: they were enlarged with ruffled membranes (Fig. 3d, right panel). In contrast, macrophages maintained on filters alone were rounded and smaller (Fig. 3d, left panel). The fact that these changes occurred within 24 hours of co-culture, suggests an enteroid-induced effect. Similar to what we described above for the enteroid monolayers alone (Fig. 1g), IL-8, TGF-β1, IFN-γ, and IL-6 were detected in media from macrophage-enteroid co-cultures (Fig. 3e). TNF-α, IL-1β, IL-10 and IL-12p70 were un-detectable (<3 pg) in co-cultures and macrophage monocultures while IL-2, IL-4, and IL-13 were secreted in low quantities likely by macrophages (see Supplementary Table S1). TGF-β1 was again secreted primarily to the basolateral side. The amount produced by the MΦ-enteroid co-culture was not different than the levels produced by the enteroids or MΦ alone, which suggests TGF-β1 could derive from either cell type but without synergism or potentiation (Fig. 3e). Meanwhile, IL-8 was also detected in the macrophage-enteroid co-culture basolateral compartment (Fig. 3e) with levels exceeding those produced by the enteroid alone but significantly lower than those produced by macrophages alone. This 1) suggests that macrophages were the main contributors of IL-8 produced basolaterally in co-culture and 2) demonstrates that epithelial cells lead to the downregulation of IL-8 produced by macrophages. Similar to IL-8, IFN-γ and IL-6 levels were substantially increased in the basolateral compartment of co-cultures upon addition of macrophages, suggesting that they were the main contributors to cytokine production (Fig. 3e). Moreover, the quantity of IFN-γ present in co-culture was almost 3 times lower as compared to level released by MΦ alone.

To further determine whether phagocytic cells could influence intestinal epithelial cell morphology, macrophage-enteroid co-cultures were established by culturing macrophages with either ND or DF intestinal enteroid monolayers (Fig. 3f, right panels). Cell height measurements of fluorescent confocal microscopy images (Fig. 3f,g) revealed a significant increase of epithelial cell height in ND and DF monolayers in the presence of macrophages as compared to ND and DF monolayers alone. Likewise, the TER measured in cultures was significantly greater when MΦ where present (Fig. 3h). These findings suggest a potential role of macrophages in enhancing the maturation of the intestinal epithelium and thickening the physical barrier^26^.

Taken together, these results demonstrated the feasibility of establishing reproducible and functional macrophage-enteroid co-cultures consisting of human primary cells and revealed the interplay of these cells and its influence on structural, physiological, and immune response features as characterized by changes in maturation, barrier function, and cytokine production.

### Bacterial infection promotes molecular and phenotypic responses of macrophage-enteroid co-cultures

**Figure 4:**
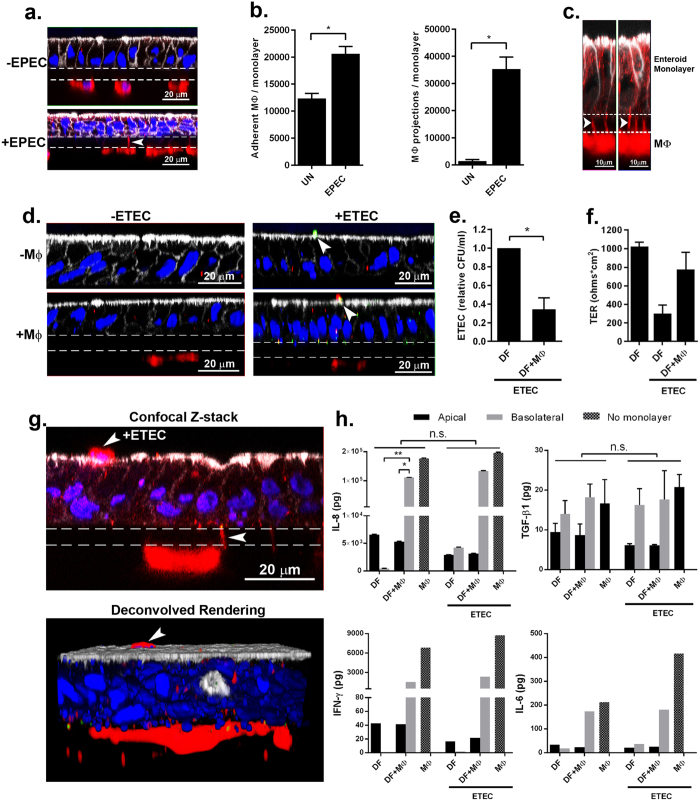
Bacterial infection of macrophage-enteroid monolayer induces morphological and physiological cell changes. (**a**) MΦ projections extended through Transwell filters into DF enteroid monolayers following overnight EPEC infection. Actin, white; nuclei, blue; CD14 (MΦ), red; filter, dashed lines. (**b**) Adherent MΦ (left panel; *indicates *p* = 4.42 × 10^−5^) and number of MΦ projections (right panel; *indicates *p* = 2.33 × 10^−9^) increased upon apical overnight EPEC infection of co-cultures. UN, uninfected. (**c**) High resolution immunofluorescence confocal microscopy showed MΦ projections (arrowheads) going through permeable support and along lateral epithelial membranes upon overnight apical EPEC infection. Actin, white; CD14 (MΦ), red; filter, dashed lines. (**d**) ETEC adhered to DF enteroid monolayers alone (upper right panel) and co-cultured with MΦ (bottom right panel) following 3 h of infection. Uninfected cultures are represented in left panels. Actin, white; nuclei, blue; ETEC, green; CD14 (MΦ), red; filter, dashed lines. (**e**) Relative number of viable ETEC collected from the enteroid monolayer following 16 h of infection decreased in the presence of MΦ. *Indicates *p* = 0.0332. (**f**) DF enteroid monolayers infected with ETEC for 16 h displayed reduced TER values that were partially restored by the presence of MΦ in the co-culture. (**g**) Confocal Z-stack image (upper panel) and derivative 3D representation (lower panel) of MΦ extending projections through the membrane to reach the apical surface of DF epithelium infected 3 h with ETEC (arrowheads). Actin, white; CD14 (MΦ), red; nuclei, blue; filter, dashed lines. (**h**) Overnight ETEC infection did not significantly change the levels of IL-8, TGF-β1, IFN-γ, and IL-6 secreted by the cultures. IL8: *indicates *p* = 0.0447; **indicates *p* = 0.0455. Data are presented as the Mean + SEM of three independent experiments involving enteroids generated from three different donors with the exception of IFN-γ and IL-6 production that represent Mean + SEM of two independent experiments with enteroids generated from two different donors.

Lastly, we assessed the suitability of the human macrophage-enteroid model to study host-pathogen interactions using ETEC and EPEC strains as model human small intestinal pathogens. Macrophages and DF enteroid monolayers were allowed to equilibrate for 24 h in co-culture, and then mimicking *in vivo* luminal exposure, ETEC or EPEC (multiplicity of infection (MOI) = 50) were applied to the apical side of enteroid monolayers in the presence or absence of MΦ. Confocal imaging of uninfected or EPEC-infected co-cultures showed the presence of MΦ projections stretching across the permeable filter, in particular upon EPEC infections (Fig. 4a). The quantitative analysis of these macrophage projections demonstrated a significant increase in their numbers as a result of EPEC infection (Fig. 4b, right panel). A similar increase in cell membrane projections was observed when MΦ were exposed to bacteria alone (Fig. 2c,d). EPEC infection was not only associated with increased projections but also with increased number of adherent macrophages (Fig. 4b, left panel) as compared to uninfected (UN) enteroid co-cultures, which suggested that luminal microbial exposure stimulated both retention of macrophages as well as their activation and response. To further localize the MΦ projections, we conducted high-resolution AIRY scan confocal microscopy of EPEC infected macrophage-enteroid co-cultures. The projections were seen extending from underneath the permeable filter and along the lateral membranes of DF enteroid monolayers (Fig. 4c, arrowheads). We further investigated these macrophage morphological changes in macrophage-enteroid co-cultures infected with ETEC. Confocal analyses showed ETEC attached to the apical side of enteroid monolayers (Fig. 4d, arrowheads). Noteworthy, the macrophages seeded underneath the filter in the macrophage-enteroid co-culture physically interacted with ETEC on the apical surface as indicated by yellow pseudocolor merge of red signal, which marks CD14^+^ macrophages, and green signal, designating ETEC (Fig. 4d, lower right panel, arrowheads). The quantification of bacteria recovered from cultures infected for 16 h demonstrated a significant decrease in viable ETEC number when macrophages where present as compared to DF enteroid monolayer alone (Fig. 4e). Interestingly, the ability of MΦ to kill apical ETEC was already visible after 30 min of infection (data not shown). Also, we noticed a rapid decrease in TER values upon overnight ETEC infection of DF enteroid monolayer, suggesting a loss of barrier function. This seemed to be partially prevented by the presence of macrophages in co-culture (Fig. 4f). Similar to EPEC infection, we found evidence of macrophage projections reaching across enteroid monolayers and trapping ETEC attached on the luminal side of the enteroid monolayer as shown by confocal microscopy and deconvolution 3D reconstruction of ETEC infected co-cultures (Fig. 4g, arrowheads). Finally, overnight infection did not trigger significant changes in production of TGFβ-1, IL-8, IFN-γ, and IL-6 by the co-culture (Fig. 4h).

In aggregate, the results show that macrophages facing the basolateral side of the epithelial cells in our co-culture model can sense, capture, and kill luminal pathogens though appendages that extend across the enteroid monolayer without disrupting the epithelial barrier and inducing a significant pro-inflammatory micro-environment.

## Discussion

In this study we have described, for the first time, the establishment and characterization of an *ex vivo* human “mini” intestine model consisting of primary intestinal epithelial cell monolayers derived from stem cell-containing crypts and monocyte-derived macrophages. An important feature of this system is that it more closely recapitulates epithelial cell diversity and functionality^13^,^27^,^28^,^29^,^30^ of the human intestine as compared to previously published models, particularly those that employ immortalized cell lines. The macrophage-enteroid co-culture model was successfully used to interrogate epithelial cell-macrophage interactions and innate immune responses to enteric pathogens.

Different from previously described *in vitro* intestinal models that rely on cancer-derived epithelial cell lines (e.g. Caco-2 and HT-29)^7^,^8^,^31^,^32^,^33^,^34^, the macrophage-enteroid model we established was based exclusively on primary human cells. Also, unlike existing cancer cell models, the enteroids that provide the framework for the macrophage-intestinal epithelium co-culture naturally exhibit the major cell types found within intestinal epithelium *in vivo,* as shown in Fig. 1f and in previous studies^18^,^22^,^29^,^35^. They also recreate the phenotypic characteristics of the intestinal segment from which they were derived^36^. Enteroid monolayers can be generated for experimental needs by proliferation of LGR5^+^ stem cell-containing ND enteroids that are maintained (bio-banked) frozen, an important practical advantage previously met only by cell lines. Furthermore, the monolayer format, as opposed to the classical Matrigel-embedded 3D cyst culture, allows manipulations both at the apical and basolateral cell surfaces in a compartmentalized manner, thereby broadening options for treatment and collection of specimens (i.e. cells and culture media). It also improves reproducibility in evaluation of outcomes by reducing the variation in cell number and the restricted lumen volume inherent in the cysts cultures^20^,^21^,^37^. An accessible apical membrane, which can only be achieved with the monolayer format, is critical for consistent exposure of the epithelium to bacterial pathogens (e.g. ETEC and EPEC) similar to what occurs *in vivo*. Although luminal infection can be performed in enteroid cysts, this would require microinjection of bacteria into each enteroid cyst lumen, which is not only impractical, requiring injection of hundreds of enteroids to infect the equivalent number of cells seeded in the enteroid monolayer cultures (~500,000 cells/0.33 cm^2^ Transwell), but also technically challenging, as the needle puncture to access the cyst lumen can irreversibly damage the enteroid.

The macrophage-enteroid model described consists of epithelial cell monolayers cultured on the top and macrophages attached underneath a permeable Transwell filter. This design allows for the cells to be in close proximity. Both epithelial cells and macrophages remained viable and functionally active in the combined culture. Importantly, the presence of MΦ further stabilized and enhanced the maturation of the enteroid monolayers as evidenced by the increased epithelial cell height and TER (Fig. 3f–h)^26^. Not only the epithelial cells changed their morphology but also the MΦ experienced visible morphological changes in the co-culture; they appeared noticeably enlarged and exhibited ruffled edges (Fig. 3d). The phenotype of these macrophages is being examined to further understand their potential effector functions. The normal human intestine contains resident anergic macrophages that contribute to maintaining immune tolerance^38^,^39^. Following intestinal infection and/or injury, blood-derived monocytes infiltrate the mucosa and differentiate into “responsive” macrophages^38^. Two major effector macrophage populations known as M1 and M2, and responsible for driving Th1 or Th2 responses (depending on the stimuli, timing, and environment, among other factors), have been described based on animals and *in vitro* studies^40^. Studies to elucidate the contribution of the intestinal epithelium in programming macrophages to polarize towards a resident, M1 or M2 phenotype are ongoing using the human enteroid–macrophage co-culture system.

Enteroids and colonoids are reliable human models for the study of viral and bacterial pathogenesis^14^,^20^,^21^,^41^,^42^. In the present study, we increased the cellular complexity by integrating macrophages to enteroid monolayers with the purpose of interrogating host-pathogen interactions and innate immune response. This was achieved by exposing a small intestinal monolayer-macrophage co-culture to EPEC and ETEC as representative human enteric pathogens^43^,^44^. Macrophages were able to physically sense and interact with *E. coli* applied to the apical side of the epithelial cells (Fig. 4d,g) and as a result, they exhibited physiological changes (Fig. 4a,c,g) consistent with the activation and deployment of phagocytic machinery and carried out bacterial killing (Fig. 4e). Furthermore, the presence of MΦ tended to prevent the increase of permeability induced by bacterial infection (Fig. 4f). Interestingly, apical *E. coli* infection improved the adherence of macrophages (Fig. 4b, left panel) and promoted the development of cell membrane projections (Fig. 4b, right panel) that extended across the epithelium and captured the organism (Fig. 4a,c,g). This phenomenon has been largely observed for human dendritic cells co-cultured with epithelial cell lines^45^,^46^,^47^ and it has been hypothesized that human macrophages could have this capacity based on studies in mice^48^,^49^,^50^. However, there was no direct evidence that this would apply to human macrophages. Our results are, to our knowledge, the first demonstration of human macrophage appendages developing and extending across a human primary intestinal epithelial cell barrier to sample luminal bacteria. Another important finding from our study was the successful bacterial attachment to differentiated (as opposed to non-differentiated) enteroid monolayers, which suggests a more efficient or specific recognition of component(s) or molecule(s) from “villus-like” cells by these enteric pathogens. Similarly, we observed a differentiated epithelium-restricted infection in human small intestinal enteroids exposed to rotavirus and human colonoids exposed to enterohemorrhagic *E. coli* (EHEC)^21^,^41^.

The analysis of cytokines in the macrophage-enteroid model revealed polarized synthesis of TGF-β1, IL-8, and IFN-γ (Figs 1g and 3e) by the epithelial cells alone or in the presence of macrophages, and IL-6 by the macrophage-enteroid co-culture (Fig. 3e). TGF-β1 and IL-8 were abundantly produced by the epithelium itself and, interestingly, are critical cytokines constitutively produced by the human intestinal mucosa and involved in mononuclear cell recruitment^25^. TGF-β1 was released primarily to the basolateral compartment regardless of epithelial cell differentiation status, which is consistent with its role of ensuring extracellular matrix maintenance and development of regulatory T cells^51^. TGF-β1 was produced in similar amounts by the enteroid cells alone, macrophages alone, or both combined, denoting no additional or synergistic effect. Interestingly, the non-differentiated monolayers secreted much higher levels of TGF-β1 than the differentiated tissue, which we suspect could be due to a positive feedback of Wnt3A, a component necessary for sustained growth of LGR5^+^ stem cells^15^ that is present in the basal (NDM) but not the differentiated media (DFM). This is in agreement with a recent report that attributed increased expression of TGF-β1 by rat hepatic cells to Wnt stimulation^52^. In all instances, the TGF-β1 was released in its inactivated form, possibly due to the absence of extracellular matrix known to be a TGF activating factor^53^.

The production of IL-8 and IFN-γ exhibited a different pattern. They were mainly released from the apical side of the enteroid monolayer, produced more abundantly by differentiated enteroid monolayers and released at remarkably higher levels to the basolateral side of the enteroid-macrophage co-culture (Figs 1g and 3d). Also, the release of IL-6, which was not polarized in enteroid mono-culture, showed a similar pattern in co-culture. Macrophages alone produced higher levels of IL-6, IL-8, and IFN-γ than the enteroids (Fig. 3e) and are presumably the main contributors of these cytokines produced by the co-culture. Interestingly, significantly less IL-8 was released in the basolateral co-culture as compared to macrophage alone. Although not significant, a similar pattern was observed for IFN-γ production (Fig. 3e). The decrease of pro-inflammatory cytokine production upon co-culture suggests a synergism between the cell types promoting the macrophage to acquire an anergic phenotype^38^. The lack of an increase in pro-inflammatory mediator production upon ETEC infection (Fig. 4h) and the high phagocytic capacity of macrophages (Fig. 4e) further sustain this hypothesis^38^. The capacity of intestinal cells to promote an anergic polarization of macrophages has been suggested in previous studies that used intestinal cell lines^31^. Further characterization of co-cultured macrophages, including their phenotype and other effector functions in steady state and during infection is warranted to better understand their functional phenotype and contribution to host homeostasis and immune defenses. The ability of the macrophage-enteroid model to detect changes and compartmentalization of immune mediators including important cytokines known to be produced into the gastrointestinal mucosa further demonstrates its accurate closeness of gut function *in vivo*.

The results our study may provide insight into how the intestinal mucosa clears pathogenic bacteria following acute infection. In humans, EPEC and ETEC infections cause gastroenteritis which resolves without medication usually within a few days post infection^54^. It is believed that pathogen clearance occurs from normal loss of infected differentiated intestinal epithelial cells since the turnover of the intestinal epithelium is 5-7 days^29^. Our findings suggest a supportive role of intestinal macrophages in bacterial clearance (i.e. increase of epithelium proximity as suggested by macrophage retention to the filter and high phagocytic activity [Fig. 4b]) and no increase in secretion of pro-inflammatory cytokines [Fig. 4h]) to promote a broad immune response.

The macrophage-enteroid model is envisioned to provide a modular format in which different (single or multiple) immune cells could be added to the epithelial cell monolayers, allowing for studies to be performed in a reductionist or step-wise approach, and in a controlled manner. The demonstration of successful co-culture of mature macrophages with enteroid monolayers produced from small intestinal and colonic intestinal sections extends its use to examine segment-specific questions (Fig. 3c). This co-culture setting will enable inclusion of epithelial and immune cells from the same subject, supporting personalized treatment studies^15^. The ability of human macrophage-enteroid to recapitulate cell identity, cytokine signaling, and response to infection support its use as an *ex vivo* model for studies that require modeling of the human gut. Its multiple improvement features make it unique and far superior than methods using human cell lines considered up to now state of the art for the study of cellular and molecular events occurring at human mucosal surface^49^,^55^. More work is currently being performed to include additional immune cell lineage, expand its characterization and identify additional readouts. This modular format also allows for further improvements such as addition of other cell lineages constitutive of the lamina propria (e.g. fibroblasts, nerves).

In conclusion, the macrophage-enteroid model described represents a practical and reliable tool to elucidate human innate immunological processes and cellular communications, and to interrogate host-pathogen interactions with a faithful representation of what would occur in the human intestinal epithelium. Furthermore, this macrophage-enteroid system provides a versatile tool to evaluate host responses to preventive or therapeutic treatments.

## Methods

### Monocyte isolation and macrophage differentiation

Human peripheral blood was collected in EDTA tubes (BD Vacutainer) from healthy volunteers provided informed consent at the University of Maryland. All experimental protocols were approved by the Institutional Review Board of the University of Maryland (IRB #HP-00040025) and all methods were carried out in accordance with the approved guidelines and regulations. Peripheral blood mononuclear cells (PBMC) were obtained by centrifugation over Ficoll-Paque PREMIUM (GE Healthcare Bio-Sciences AB, USA) and residual red blood cells were lysed by treatment with ACK (Ammonium-Chloride-Potassium) Lysing Buffer (Quality Biological, USA). Monocytes were enriched from PBMC by using the Pan Monocyte Isolation Kit (Miltenyi Biotec, USA), according to the manufacturer’s instructions. For macrophage differentiation, cells were seeded in 6- or 12-well plates, at 10^6^ cells/ml, and incubated for 6 days in RPMI (Gibco/Life Technologies, USA) supplemented with 10% heat-inactivated fetal bovine serum (FBS) (HyClone Laboratories, USA), MEM non-essential amino acids 1X, 1 mM sodium pyruvate and 55 μM 2-Mecaptoethanol (Gibco, Life Technologies), Penicillin/Streptomycin (HyClone Laboratories, USA) and 50 ng/ml Macrophage Colony-Stimulating Factor (M-CSF) (BioLegend, USA). Additional M-CSF was added on day 2. Complete media was changed on day 4. Cells were maintained at 37 °C in 5% CO_2_.

### Tissue collection and enteroid generation

Human enteroid cultures were established from biopsies obtained after endoscopic or surgical procedures utilizing the methods developed by the laboratory of Dr. Hans Clevers^22^. De-identified biopsy tissue was obtained from healthy subjects provided informed consent at Johns Hopkins University and all methods were carried out in accordance with approved guidelines and regulations. All experimental protocols were approved by the Johns Hopkins University Institutional Review Board (IRB# NA_00038329). Briefly, enteroids generated from isolated intestinal crypts^17^ were maintained as cysts embedded in Matrigel (Corning, USA) in 24-well plates and cultured in Wnt3A containing NDM. Once multiple enteroid cultures had been generated, multiple wells were pooled, triturated in Cultrex Organoid Harvesting Solution (Trevigen, USA), and the fragments were collected by centrifugation and resuspended in NDM. Enteroid fragments (100 μl) were added onto 0.4 μm or 1.0 μm pore transparent polyester (PET) membrane 24-well cell culture inserts (Transwell; Corning, USA or Millipore, USA) pre-coated with human collagen IV (30 μg/ml; Sigma-Aldrich, USA). NDM (600 μl) was added to the wells of the receiver plate, and the cultures incubated at 37 °C, 5% CO_2_. Under these conditions, enteroid cultures reached confluency in 7-14 days. Monolayer differentiation was induced by incubation in Wnt3A-free^20^ and Rspo-1-free DFM for five days. Monolayer confluency and differentiation were monitored by measuring TER with an ohmmeter (EVOM^2^; World Precision Instruments, USA). The unit area resistances (ohm*cm^2^) were calculated according to the growth surface area of the inserts; 0.33 cm^2^ for both 0.4 and 1.0 μm inserts. Enteroids were derived from segments of the small intestine (duodenum, n = 5; jejunum, n = 1) and proximal colon (n = 1) for Fig. 3c. For all other experiments, results were generated from duodenal and jejunal enteroids derived from intestinal biopsies of healthy subjects.

### Enteroid monolayer and macrophage co-culture establishment

ND or DF enteroid monolayers seeded on inserts were inverted and placed into an empty 12-well plate. Fifty microliters of NDM or DFM supplemented with 10 ng/ml of M-CSF and containing 10^5^ monocyte-derived macrophages (MΦ) were added onto the bottom surface of the inserts, and cells were allowed to attach for 2 h at 37 °C in 5% CO_2_. The inserts remained wet throughout the MФ adherence period. After this incubation, the inserts were turned back to their original position into a 24-well receiver plate and NDM or DFM supplemented with M-CSF (10 ng/ml) was added into the insert (100 μl) and into the well (600 μl). TER measurements indicated as 0 h were collected prior to preparing the co-culture.

### *Escherichia coli* strains and infections

ETEC H-10407^56^,^57^ and EPEC E2348/69^58^ were generously provided by James Kaper (University of Maryland, Baltimore, USA). Both strains were grown from frozen stocks (−80 °C) at 37 °C on Luria broth (LB) agar plates 2-3 days prior to experiments. The day before infections, single colonies were inoculated in 2 ml of Advanced DMEM/F12 tissue culture medium (Gibco/Life Technologies, USA) and grown overnight with vigorous shaking at 37 °C. The day of the infections, the *E. coli* cell density of the overnight culture(s) was adjusted to 10^9^ CFU/ml in Advanced DMEM/F12, and 5 μL (5 × 10^6^) was added to MΦ, monolayers or 24 h co-cultures to achieve a multiplicity of infection of 50 relative to MΦ (10^5^). *E. coli* infections were allowed to progress for 30 min, 3 h, overnight (14 h–16 h), or 24 h.

### ETEC killing assays

The ability of MΦ in co-culture to kill ETEC was measured by bacterial killing assay. Enteroid monolayers alone or co-cultured with MΦ for 24 h were infected apically with ETEC (infection doses ranged from 5 × 10^6^ to 10 × 10^6^ CFU) for 30 min or overnight. After infection, the monolayers were washed 3 times with sterile PBS (phosphate buffered saline; 137 mM NaCl, 2.7 mM KCl, 8 mM Na_2_HPO_4,_ 1.5 mM KH_2_PO_4_, pH 7.3) and lysed in a solution of 0.5% sodium deoxycholate (Sigma-Aldrich) by applying vigorous vortexing. The lysate was serially diluted in sterile PBS and spread onto Luria Broth agar plates. CFU were counted after overnight incubation at 37 °C. Results for 16 h infection were obtained from three different small intestinal lines (n = 3). Data are presented as the relative change in the number of viable bacteria observed in ETEC-infected DF macrophage-enteroid monolayer co-cultures compared to DF enteroid monolayers alone exposed to ETEC.

### Immunofluorescence staining

Human enteroid monolayers and Mɸ were fixed in aqueous 4% paraformaldehyde (PFA; Electron Microscopy Sciences, USA) for at least 30 min at room temperature (RT) and then washed with PBS for 10 min at RT. Permeabilization and blocking were carried out simultaneously in a solution of 15% FBS, 2% BSA, and 0.1% saponin (all Sigma-Aldrich, USA) in PBS for 30 min at RT. Cells were rinsed with PBS and incubated overnight at 4 °C with primary antibodies diluted 1:100 in PBS containing 15% FBS and 2% BSA. Primary antibodies included Rabbit anti-Muc2 (Sigma-Aldrich, USA), Rabbit anti-phospho-Ezrin (Abcam, USA), Rabbit anti-lysozyme (DAKO, USA), Mouse anti-CD14 (Abcam, USA), Mouse anti-chromogranin A (The Clinical for Proteomic Technologies for Cancer; National Cancer Institute, NIH, USA) and rabbit sera containing anti-colonization factor 1 (CFA/I) for ETEC and anti-H6 flagellin for EPEC (provided by James Kaper). Because EPEC flagella are lost soon after adhesion to Caco-2 monolayers^59^, EPEC were also visualized by DNA staining in the absence of H6 staining (Fig. 2c). Stained cells were then washed 3 times for 10 min each with PBS followed by secondary antibodies diluted 1:100 in PBS. Secondary antibodies included Goat anti-Rabbit-Alexa-488, Goat anti-Mouse-Alexa-488, Goat anti-Rabbit-Alexa-568, Goat anti-Mouse-Alexa-568 (all from Molecular Probes/Invitrogen, USA). Probes included wheat germ agglutinin (WGA-488) and phalloidin (568 or 633) (Life Technologies). Hoechst (Vector Laboratories, USA) for nuclear/DNA labeling was used at a 1:1000 dilution in PBS. After incubation, cells were washed 3 times for 10 min each and mounted in ProLong Gold (Vector Laboratories, USA) overnight at 4 °C.

### Confocal Microscopy

Confocal imaging was carried out at the Fluorescence Imaging Core of the Hopkins Basic Research Digestive Disease Development Center using an LSM-510 META laser scanning confocal microscope (Zeiss, Germany) running ZEN 2012 (black edition) imaging software (Zeiss, Germany). All images were captured with a 40X oil objective. For qualitative analysis, image settings were adjusted to optimize signal. For quantitative analysis, the same settings were used to image across samples (e.g. for enumeration of MΦ or MΦ projections). Deconvolved rendering of confocal fluorescent image data were obtained using Volocity 3D Image Analysis Software (v6.1, PerkinElmer; USA) (Fig. 4g, bottom panel). The optical section (0.159 μm) shown in Fig. 4c was generated on an LSM-880 with AIRY scan detector and processor running ZEN 2.1 (black edition) under a 63X oil objective. Images were minimally manipulated using either ZEN 2012 or Photoshop (version CS3, Adobe) to emphasize macrophages and bacteria staining. Signal processing was applied equally across the entire image.

### Flow cytometry

The following human specific monoclonal antibodies were obtained from BD PharMingen, USA: 2331/FUN-1 (anti-CD86, FITC-conjugated), 10.1 (anti-CD64, FITC-conjugated), M5E2 (anti-CD14, APC-conjugated), G46-6 (anti-HLA-DR APC- and V450-conjugated), L307.4 (anti-CD80, PE-Cy7-conjugated), and 3G8 (anti-CD16, PE-Cy7-conjugated). K0124E1 (anti-human CX_3_CR1, PE-conjugated), 10.1 (anti-CD64, PerCP/Cy5.5-conjugated), and A59 (anti-human CD89, PE-Cy7-conjugated) were obtained from BioLegend, USA. Appropriate isotype-matched control antibodies were purchased from the same manufacturers and used at the same concentration as their respective specific antibody. Cells were blocked in 2% normal mouse serum (Invitrogen, Frederick, MD, USA) for 15 min at 4 °C. After washing, 10^5^ to 10^6^ cells were resuspended in 100 μl of PBS containing 0.5% BSA and 2 mM EDTA (all Sigma-Aldrich) and were incubated with antibodies for 30 min at 4 °C. Cells were then washed 3 times in staining buffer and either analyzed or fixed in 4% PFA for 15 min at 4 °C. Fixed cells were maintained up to 3 days at 4 °C before acquisition. Cells were analyzed in a Guava 8HT using Guava ExpressPro software (Millipore USA) or BD LSR Fortessa using FACSDIVA software (BD Biosciences, USA). Data were analyzed using FlowJo software (v10.1). Fluorescence minus one (FMO) or alternatively beads (OneComp Beads, eBiosciences) were used for compensation. Cells were counted using Guava Viacount Flex Reagent (Millipore, USA) according to the manufacturer’s instruction and analyzed on a Guava 8HT using Guava ViaCount software (Millipore, USA).

### Cytokine and chemokine measurements

Cytokine and chemokines were measured by electrochemiluminescence microarray using commercial assays (Meso Scale Diagnostic, Rockville, MD) following the manufacturer’s instructions. IL-8, IL-1β, IL-2, IL-4, IL-10, IL-13, IL-12p70, TNF-α, IFN-γ, IL-6, and TGF-β1 were reported as pg contained in the total volume of culture supernatant obtained from the apical and basolateral compartments of the enteroid or enteroid-MΦ co-culture, or from the MΦ cultured alone at the specified times.

### Statistics

Statistical significances were calculated using the Student’s *t*-test or alternatively Mann–Whitney–Wilcoxon test in absence of normal distribution of the values. TER and cell height values from ND or DF monolayers, cytokine/chemokine values from different conditions, and apical and basolateral compartments from ND or DF were considered as paired for *p* calculation. Cytokine/chemokine production by ND and DF enteroids, number of MΦ, and MΦ projection values were considered as unpaired for *p* calculation. Unequal variance was considered for ETEC CFU value comparisons. Statistical significances were assessed to compare groups including minimum n = 3 replicates. Exact *p* values are indicated in Figure legends.

## Additional Information

**How to cite this article:** Noel, G. *et al*. A primary human macrophage-enteroid co-culture model to investigate mucosal gut physiology and host-pathogen interactions. *Sci. Rep.* **7**, 45270; doi: 10.1038/srep45270 (2017).

**Publisher's note:** Springer Nature remains neutral with regard to jurisdictional claims in published maps and institutional affiliations.

## Supplementary Material

Supplementary Table 1
