# Erratum: A primary human macrophage-enteroid co-culture model to investigate mucosal gut physiology and host-pathogen interactions

**DOI:** 10.1038/srep46790

**Published:** 2017-05-03

**Authors:** Gaelle Noel, Nicholas W. Baetz, Janet F. Staab, Mark Donowitz, Olga Kovbasnjuk, Marcela F. Pasetti, Nicholas C. Zachos

Scientific Reports 7: Article number: 4527010.1038/srep45270; published online: 03 27 2017; updated: 05 03 2017

The previous PDF version of this Article contained a typographical error in the Results section under the subheading ‘Generation of mature human macrophages to establish a macrophage-enteroid co-culture system’. The text had been inadvertently formatted incorrectly in the PDF and now reads:

“On the basis of cell surface marker expression that included: a major CD14+”.

In addition, the Acknowledgements section in this Article was incomplete:

“The authors wish to acknowledge the Integrated Physiology Core and the Imaging Core of the Hopkins Conte Digestive Disease Basic and Translational Research Core Center. They also want to thank Robin Barnes, a Clinical Research Nurse at University of Maryland for her assistance in obtaining peripheral blood samples and Dr. James Kaper (University of Maryland School of Medicine) for kindly providing EPEC and ETEC strains and antibodies. In addition, the authors wish to thank Alma Arnold and Glenn Doherty from Carl Zeiss Microscopy (Thornwood, NY) for capture and AIRY scan processing of image data presented in Fig. 4c. This work was funded by Grand Challenge Exploration award OPP1118529, P30 DK-089502 and P01-AI125181”.

Now reads:

“The authors wish to acknowledge the Integrated Physiology Core and the Imaging Core of the Hopkins Conte Digestive Disease Basic and Translational Research Core Center. They also want to thank Robin Barnes, a Clinical Research Nurse at University of Maryland for her assistance in obtaining peripheral blood samples and Dr. James Kaper (University of Maryland School of Medicine) for kindly providing EPEC and ETEC strains and antibodies. In addition, the authors wish to thank Alma Arnold and Glenn Doherty from Carl Zeiss Microscopy (Thornwood, NY) for capture and AIRY scan processing of image data presented in Fig. 4c. This work was funded by Grand Challenge Exploration award OPP1118529 and National Institutes of Health awards P30-DK089502 and P01-AI125181”.

These errors have now been corrected in the PDF and HTML versions of this Article.

