# Supplementary material for: A primary human macrophage-enteroid co-culture model to investigate mucosal gut physiology and host-pathogen interactions
Source: Sci Rep. 2017 Mar 27;7:45270. doi: 10.1038/srep45270 (PMC5366908; doi:10.1038/srep45270)
Supplement: Supplementary Table 1 [file srep45270-s1.pdf]

A primary human macrophage-enteroid co-culture model to investigate mucosal gut physiology and host-pathogen interactions

Gaelle Noel, Nicholas W. Baetz, Janet F. Staab, Mark Donowitz, Olga Kovbasnjuk, Marcela F. Pasetti, Nicholas C. Zachos

SUPPLEMENTARY INFORMATION

	DF		DF + Mφ		Mφ
	Apical	Basolateral	Apical	Basolateral	
**TNF-α**	0.0 ± 0.0	0.1 ± 0.0	0.0 ± 0.0	0.3 ± 0.0	0.5 ± 0.1
**IL-1β**	0.1 ± 0.0	0.1 ± 0.1	0.1 ± 0.0	1.3 ± 0.3	3.0 ± 0.5
**IL-2**	0.1 ± 0.0	0.4 ± 0.1	0.1 ± 0.0	10.6 ± 4.0	15.0 ± 1.8
**IL-4**	0.0 ± 0.0	0.0 ± 0.0	0.0 ± 0.0	61.4 ± 30.2	48.1 ± 19.7
**IL-10**	0.0 ± 0.0	0.0 ± 0.0	0.0 ± 0.0	0.8 ± 0.3	1.7 ± 0.3
**IL-12p70**	0.0 ± 0.0	0.0 ± 0.0	0.0 ± 0.0	0.1 ± 0.0	0.2 ± 0.1
**IL-13**	0.1 ± 0.1	0.0 ± 0.0	0.2 ± 0.1	6.8 ± 2.3	15.6 ± 4.4

Supplementary Table S1: Human macrophage-enteroids discretely secrete additional cytokines *ex vivo.*

Total amount of IL-1β, IL-2, IL-4, IL-10, IL-12p70, IL-13, and TNF-α released in the apical and basolateral media of DF enteroid monolayer in absence or presence of macrophages (MΦ), and by MΦ alone following 24h of culture. Values are presented as Mean ± SEM of 3 independent experiments involving 3 different small intestinal enteroid lines.
